# Current Trends in the Production of Probiotic Formulations

**DOI:** 10.3390/foods11152330

**Published:** 2022-08-04

**Authors:** Jakub Kiepś, Radosław Dembczyński

**Affiliations:** Department of Biotechnology and Food Microbiology, Poznań University of Life Sciences, Wojska Polskiego 48, 60-627 Poznań, Poland

**Keywords:** lactic acid bacteria, spray drying, freeze drying, vacuum drying, fluid bed drying, viability, shelf-life, stress factors, protectants

## Abstract

Preparations containing probiotic strains of bacteria have a beneficial effect on human and animal health. The benefits of probiotics translate into an increased interest in techniques for the preservation of microorganisms. This review compares different drying methods and their improvements, with specific reference to processing conditions, microorganisms, and protective substances. It also highlights some factors that may influence the quality and stability of the final probiotic preparations, including thermal, osmotic, oxidative, and acidic stresses, as well as dehydration and shear forces. Processing and storage result in the loss of viability and stability in probiotic formulations. Herein, the addition of protective substances, the optimization of process parameters, and the adaptation of cells to stress factors before drying are described as countermeasures to these challenges. The latest trends and developments in the fields of drying technologies and probiotic production are also discussed. These developments include novel application methods, controlled release, the use of food matrices, and the use of analytical methods to determine the viability of probiotic bacteria.

## 1. Introduction

Concentrated probiotic bacteria used in animal nutrition and consumed by humans most commonly occur in the form of dried biomass. Most probiotic bacteria belong to the group of lactic acid bacteria with GRAS status. Probiotic preparations come in various forms: capsules, suspensions, powders, and combined into probiotic food. All of these forms could be further improved as they share the issue of a loss of viability during both processing and storage. For example, fluid suspensions, however relatively easy to produce, are the least stable form of probiotics with the shortest shelf-life [1]. Probiotics in solid forms, such as capsules and powders, are more stable and can be stored for a longer period of time than fluid suspensions. Their viability and shelf-life could be further improved by the addition of protective substances or by the introduction of stress factors to probiotic bacteria prior to their drying. All probiotics contain live bacteria, mainly from the genera *Lactobacillus* and *Bifidobacterium* [2]. The results of clinical trials show the positive effects of taking probiotics on diseases of the gastrointestinal tract, including irritable bowel syndrome, diarrhea, enteritis, and allergic conditions, such as atopic dermatitis. Probiotics have also been shown to increase the body’s immune resistance through immunomodulation [3]. As described by the WHO, to have a beneficial health effect, probiotic preparations should contain a minimum number of live bacteria (colony-forming units), i.e., at least 10^6^ cfu/g. The qualitative parameters of dry cells (the number of live cells and their biological activity) usually depend on the applied drying method. These methods include spray drying, freeze drying, vacuum drying, and fluid bed drying. The obtained probiotic formulations can then be used in a variety of novel formulations, such as nasal sprays, creams, and lotions [4,5]. They are also used in food matrices to enrich their beneficial health effects. Some food matrices, e.g., ice cream, are also used to improve the stability and shelf-life of probiotics [6].

## 2. Drying Methods

**Figure 1:**
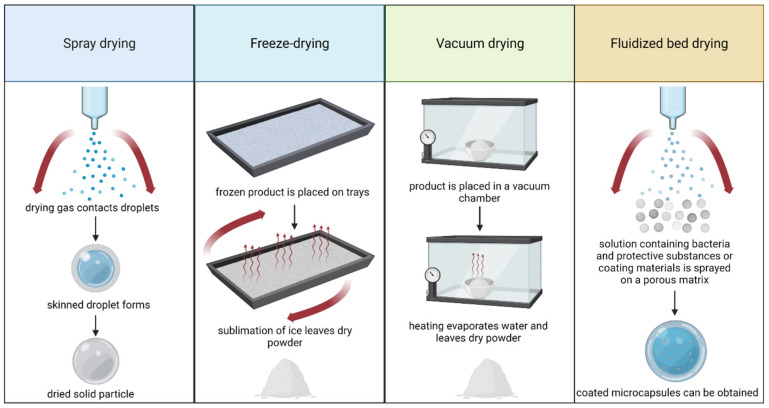
Summary of the basic principles for the most commonly applied drying techniques. Created with Biorender.com (accessed on 27 July 2022).

Cryopreservation is one of the most commonly used methods of preserving and storing live cultures of microorganisms for a long time; this approach is used in microbiological laboratories. From a commercial point of view, this method has disadvantages, which include high levels of energy consumption and the need to maintain and transport the samples at temperatures below zero. In addition, freezing and thawing the cells of microorganisms can damage them. When large amounts of probiotic cultures are produced, it is preferred to use other methods of preservation, such as different drying techniques [7]. The basic principles of these drying techniques are summarized in Figure 1. Examples of the viability of some probiotic strains dried by different methods are shown in Table 1, and the protective effects of different materials are summarized in Table 2.

### 2.1. Traditional Drying Methods

#### 2.1.1. Spray Drying

Spray drying is a fast and relatively inexpensive technique that makes it possible to obtain dry, mostly spherical powder particles with good flow properties, uniform shape, and particle size distribution [8]. The drying process occurs in four stages. In the first stage, the microorganism suspension is sprayed into small droplets. Then, the droplets are carried by hot air, with three different methods for the air to contact the droplets: co-current, counter-current, and mixed [9]. Because probiotic bacteria are sensitive to high temperatures, the co-current flow is usually applied. In this way, drops with a high water content contact the high-temperature inlet air, and the dry particles contact the lowest-temperature exhaust air, which reduces the risk of damage to microorganisms. For example, in previous works, inlet air temperatures for the spray drying of *L. rhamnosus* were 130–150 °C [10]; for probiotic almond milk powder containing *L. plantarum*, temperatures of 170–190 °C were used [11]. The third stage of the spray-drying process is the drying of the droplets and the formation of dry particles. It is at this stage that the microorganisms are most susceptible to thermal inactivation [12].

In comparison with other methods, spray drying has several advantages. These include a short drying time, the capacity for continuous operation, and low cost; these factors translate into the possibility of drying large volumes of suspension in a relatively short time. Additionally, it is possible to influence the characteristics of the powder, and the process is relatively easy to scale up [13].

Spray freeze drying is a method that combines the advantages of spray drying and freeze drying. It is conducted in three stages: the atomization of a cell suspension in a spray dryer, freezing (over liquid nitrogen), and freeze drying. In this process, the cells are first atomized with the addition of a protective material (such as WPI) over liquid nitrogen, which allows the droplets to quickly freeze. They are then additionally freeze dried. Studies conducted using spray freeze drying showed an encapsulation efficiency of 88–95% for *L. plantarum* MTCC 5422 with various wall materials. The overall encapsulation efficiency was, however, lower than in regular freeze drying due to the additional stress factors that occurred during atomization and freezing [14].

#### 2.1.2. Freeze Drying

Freeze drying, or lyophilization, is a common method of removing water from probiotic bacterial cells to ensure their storage stability. The dryer consists of a vacuum chamber with a freezing system, a system for removing water vapor, and heating elements that are necessary to supply heat for sublimation. The freeze-drying process occurs in three stages: the freezing of the cell culture, sublimation, and final drying, the first of which is often carried out outside the dryer. In the next stage of the process, the frozen water is removed in the sublimation process under reduced pressure, and in the last stage, non-frozen water is removed in the process of desorption in order to attain the final water content [15]). Because the conditions of the freeze-drying process are milder than those of the spray-drying process, probiotic cultures dried by this method usually show higher rates of survival [16].

Despite the frequent use of this method of drying microorganisms, including probiotics, freeze drying has several disadvantages. It is an expensive and lengthy batch process, and the final product is often compact and hard. Regardless of this, freeze drying is a useful and widely used drying technique with several strategies already developed to maximize the viability of probiotic cultures. Among such modifications is pulse-spouted microwave freeze drying [17,18]. It aims to shorten the drying time in comparison with the traditional variant of freeze drying.

#### 2.1.3. Vacuum Drying

A vacuum dryer consists of a chamber in which heated shelves are located. Trays containing wet biomass are placed on the shelves, and the water vapor is removed using a vacuum pump and condensed in a condenser. During freeze drying, the cells are frozen before the water is removed, while in vacuum drying, they remain in liquid form. Moreover, vacuum dryers operate at a higher temperature and pressure, and the energy consumption is 40% lower compared to freeze drying [19]. Typical pressures for vacuum drying are above 30–60 mbar, which corresponds to a boiling point of water of 25–30 °C; for freeze drying, the pressure is lower than 6 mbar [7,20]. The main disadvantage of vacuum drying, compared to spray or fluid bed drying, is the long processing time, ranging from 20 to 100 h [1].

New developments in the field of vacuum drying include the use of pulse-spouted microwave vacuum drying (PSMVD) [17]. Banana cubes dried by PSMVD showed an expansion trend, resulting in a better structure and rehydration ratio. PSMVD-dried cubes also provided better nutritional value as measured by the content of ascorbic acid, which reached 7.96 mg 100 g^−1^ (compared to 4.23 mg 100 g^−1^ for the traditional variant of vacuum drying).

#### 2.1.4. Fluidized Bed Drying

Fluidized bed drying is a process in which heated gas, usually air, flows at a certain speed through a layer of solid particles, causing them to reach a fluidized flow state. Because the fluidization process has very good heat and mass exchange conditions, water is quickly evaporated from the dried material. The time required for fluidized bed drying (1 min to 2 h) is shorter than that of freeze drying and comparable to that of spray drying. The relatively low drying temperature does not cause thermal stress [1]. The cell biomass is not dried on its own but mixed with additional material that acts as a carrier or matrix to which the cells adhere. In practice, many loose and powdered materials have been used for this purpose, such as wheat flour, skimmed milk powder, casein, maltodextrin, starch, microcrystalline cellulose, inulin, and NaCl [1,21,22,23]. Usually, the matrix material is first placed in the dryer chamber and fluidized; then, the bacterial suspension is sprayed onto the fluidized matrix via a nozzle. Another method is to prepare the granulate first; after mixing the wet biomass with the matrix material and forming the granules using a sieve, pellet mill, or drum granulator, they are then dried in a fluidized bed [24,25]. It is important to consider the purpose of the dried bacterial product when selecting the matrix material as it can have variable technological characteristics or provide additional properties (e.g., prebiotic inulin) [21].

### 2.2. Novel Immobilization Methods

**Figure 2:**
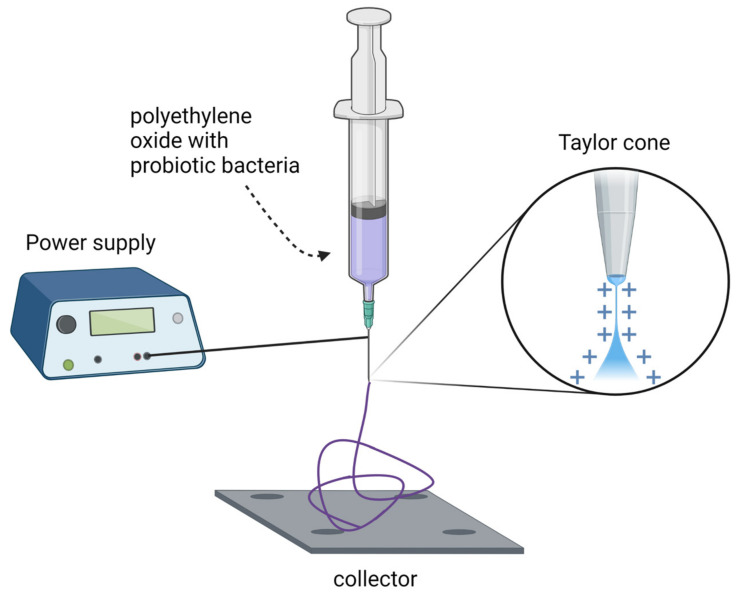
Principal elements of nanofiber fabrication by electrospinning. Created with Biorender.com (accessed on 1 June 2022).

New methods of probiotic cell immobilization are also emerging as an alternative to drying processes and microencapsulation. Škrlec et al. [26]. have developed two types of electrospun nanofibers (Figure 2): monolithic poly(ethylene oxide) and composite poly(ethylene oxide)/lyoprotectant. *L. plantarum* cells were applied to these nanofibers and achieved high cell concentrations (up to 7.6 × 10^8^ cfu/mg). Moreover, their survival during storage at 25 °C was promising, with a 1.83 log decrease in viability over 24 weeks (from 8.51 to 6.68 cfu/mg). For comparison, lyophilized samples showed a 1.70 log decrease in viability over the same period (from 8.97 to 7.27 cfu/mg). The differences between nanofibers and lyophilized samples stored at 4 °C were also minimal. The release time was also measured. Nearly all (>90%) *L. plantarum* cells were released from the nanofibers within the first 30 min of the experiment. This was confirmed by both plate cell counts and fluorescence measurements of the mCherry protein and provided insight into possible applications of probiotic-loaded nanofibers. The 30 min release period allowed for the controlled delivery of probiotic bacteria and was sufficient for their adhesion to the intestinal epithelium and mucosa.

3D printing is a novel method used in food industry applications that allows for the design of customized products. Yoha et al. [27] studied the effects of the 3D printing of probiotic encapsulates on their viability. To prepare the base for the 3D printing of *L. plantarum* (NCIM 2083), cells were dried using four encapsulation techniques (spray drying, freeze drying, spray freeze drying, and refractance window drying) with the addition of a prebiotic matrix (fructooligosaccharide, whey protein, and maltodextrin in a ratio 4:1:1, respectively). Dried microcapsules were then used for 3D printing with a composite flour formulation using the food 3D printer CARK [28]. Yoha et al. (2021) reported that the 3D printing process did not lower the viability of probiotic bacteria. They also determined that freeze drying yielded the highest level of cell viability, i.e., 8.23 ± 0.21 log_10_ CFU/g, followed by spray freeze drying (8.18 ± 0.16 log_10_ CFU/g). Under in vitro digestion, freeze-dried probiotics showed a lower level of viability (6.12 ± 0.29 log_10_ CFU/mL) than spray-freeze-dried samples (6.43 ± 0.29 log_10_ CFU/mL). 

### 2.3. Auxiliary Methods

The standard drying methods, by themselves, are well-established and optimized. They can be, however, improved upon by the introduction of auxiliary methods, such as fluid bed coating. The combination of two different techniques allows the use of the most popular methods, such as spray drying or freeze drying, in the first step; then, the stability of the preparation is improved upon by fluid bed coating with different protective substances.

In their research, Jacobsen et al. [24] showed that the viability of freeze-dried probiotics after granulation and fluid bed coating was only slightly reduced. Freeze-dried *L. reuteri* LR92 was used to produce probiotic pellets by granulation, extrusion, and spheronization. The obtained probiotic pellets were then subjected to fluid coating with the Eudragit S100 and Eudragit FS30D coating suspensions to achieve delayed release. To evaluate the targeted delivery of coated probiotic pellets, an in vitro model simulating the conditions of the human gastric system, duodenum/jejunum, and ileum was developed. The release of active substances from the coated pellets was determined by the quantification of the released marker riboflavin with HPLC. The results show that the coated probiotic pellets achieved the desired release profile (release in the ileum) based on the release of riboflavin.

Fluidized bed drying was also used to prepare dried probiotic apple snacks. The apple cubes were first dried in a fluidized bed at 50 °C until they reached a level of water activity lower than 0.5 and a moisture content below 15%. Then, the dried apple cubes were coated with a solution of hydroxyethyl cellulose and polyethylene glycol mixed with washed *Bacillus coagulans* spores. The optimal ratio of coating substances was established at 0.125 g of HEC to 11.7 uL of PEG. Coating with the optimal coating mixture resulted in achieving a 77.7% coverage of the sample area. The achieved product was microbiologically stable during storage at room temperature for 90 days and was able to maintain at least an 8 log CFU/30 g portion. Reductions in enzymatic activity, specifically the activity of polyphenol oxidase (by 86%) and of peroxidase (by 92%), represented an additional improvement [29].

### 2.4. Factors Affecting the Viability of Probiotics during Drying

**Figure 3:**
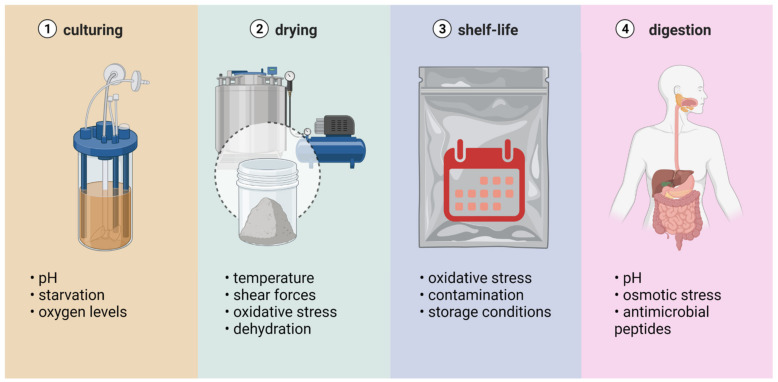
Stress factors affecting probiotics during different stages of their preparation and administration. Created with Biorender.com (accessed on 27 July 2022).

During drying, probiotic microorganisms are exposed to various stress factors, such as excessive dehydration and thermal, mechanical, osmotic, and oxidative stresses [30]. Probiotic microorganisms belong to the group of products with low thermal stability; at the same time, there is a certain critical water content that must be maintained. A reduction in water content below this critical value may cause the dehydration of the cells and, therefore, their inactivation. Thermal stress and dehydration are considered the main causes of losses of the viability of probiotic bacteria during spray drying. Stress factors that affect probiotic bacteria during different processing stages are presented in Figure 3.

Thermal stress, i.e., the heat inactivation of microorganisms, is a significant risk in the second stage of drying, according to various authors. In this phase of the process, microorganisms can reach the temperature of the drying air, especially since the dried particles often remain in the dryer until the entire process is completed. Not all bacteria are equally prone to thermal inactivation. For example, *L. acidophilus* has shown better survival rates under various drying temperatures than *E. coli* K12. This can be explained by differences in the thickness of the cell wall, which is thicker in the case of Gram-positive bacteria (e.g., *L. acidophilus)*. Moreover, drying in a medium containing nutrient broth yielded better survival rates than drying in a medium without broth components [31].

High temperatures can denature intracellular proteins and destabilize cell membranes, which in turn leads to cell death. At the same time, higher temperatures cause a decrease in the water activity of the dried samples, which translates into increased storage stability. Therefore, when choosing spray drying parameters for probiotic microorganisms, it is important to determine an optimal outlet air temperature that is high enough for the dried samples to have low water activity and, on the other hand, low enough to prevent cell damage [20]. Air temperature also significantly affects the bulk density of dried probiotic powders. As the temperature increases, evaporation rates also increase. The powder dries to a more porous structure and is more prone to forming hollow particles [32].

The inactivation of microorganisms caused by dehydration often occurs simultaneously with heat damage. During drying, water molecules are removed from the cells, which limits chemical reactions and metabolic activity. Because water is essential for the stabilization of various components of the cell, its removal may result in a loss of cell integrity, changes in cell structure, and damage to the enzyme system [7]. This applies to, among other things, changes in the lipid bilayer of the cell membrane that can cause the leakage of intracellular fluid and, consequently, cell death [33]. In experiments on drying single droplets of an *L. plantarum* suspension, it was shown that at an outlet air temperature below 45 °C, inactivation due to dehydration was dominant, while above this temperature, dehydration and temperature stress occurred simultaneously [34]. The authors of the study also believed that the longer the drying time, the more cells would undergo dehydrative inactivation.

Osmotic stress during drying occurs as a result of cells losing water to the environment, which increases the molarity of the intracellular solution and reduces the volume of the cytoplasm. A loss of cellular turgor occurs, and the cell undergoes plasmolysis, which, as a consequence, leads to a loss of viability [7].

Oxidative stress is caused by oxygen contained in the air and dissolved in an aqueous suspension of microorganisms [35]. Probiotic bacterial tolerance to oxygen is varied, with most *Bifidobacterium* species requiring strict anaerobic conditions and many strains of lactic acid bacteria tolerating oxygen. Oxidative stress is caused by reactive oxygen particles interacting with proteins, nucleic acids, and lipids. As a result, protein denaturation and lipid oxidation occur, leading to cell membrane damage and cell death [30].

The cells of probiotic microorganisms can also be inactivated by shear forces as their suspension is sprayed into the dryer head. Several studies have shown a relationship between the pressure of the suspension administered through atomizing nozzles and the survival of probiotic bacteria [35].

Similar to other methods of drying probiotic bacteria, in fluidized bed drying, certain factors cause losses of cell viability, mainly by osmotic stress, excessive dehydration, and oxidative stress [20]. It is believed that the threat of thermal shock at the temperatures used in the fluidized bed drying of microorganisms is insignificant up to a material moisture level of 15% and increases as the water activity of the dried material decreases [7]. Additionally, the pressure in the atomizing nozzle can affect the viability of the cells. An increase in nozzle pressure above 1.5 bar reduced the viability of *Enterococcus faecium* cells by 4 log cycles [23].

In the process of freeze drying, microorganisms are exposed to various stress factors caused by freezing and the sublimation of ice, leading to changes in the cell. These include deformation, mechanical damage to the cellular structure by ice crystals formed during the process, the loss of semipermeable properties of cell membranes, changes in the structure of membrane lipids, and the denaturation of protein components due to an increase in the concentration of intracellular compounds [7].

Ice crystals form at the biomass-freezing stage. The crystal growth depends on the freezing rate and temperature. Rapid freezing is recommended because the ice crystals reach small dimensions and do not damage the microbial cells. In addition, slowly lowering the temperature leads to ice forming mainly outside the cells, which leads to excessive dehydration. The formation of ice crystals is not the only threat to cell viability. During ice crystallization, the intracellular solution thickens, which can lead to osmotic stress. Moreover, the removal of water bound to the cells may damage surface proteins, the cell wall, and the cytoplasmic membrane. The lipid fraction of the bilayer cell membrane, where the structure of polar phospholipid parts may change, is particularly vulnerable to damage during dehydration [33].

Because vacuum drying occurs at a temperature higher than freeze drying but lower than spray drying, it is a milder process in terms of the effects of high or low temperature on the cells of microorganisms. Furthermore, the lack of oxygen in the drying environment can reduce oxidative stress, especially when drying oxygen-sensitive bacteria, e.g., *Bifidobacteria* [30]. Dehydration stress, however, is considered a major threat to cell viability during this process. For example, during vacuum drying, cell damage is observed mainly in the cell membrane [36].

The described stress factors, when introduced in a controlled manner in the culturing stage, can also be used to prevent the loss of viability during drying and storage. Research by Hernández et al. [37] confirms that fermentation parameters, such as pH and temperature, influence the stress resilience of *L. reuteri* DSM 17,938 during freeze drying. Simultaneous exposure to mild heat (50 °C) and osmotic stress (0.6 M of NaCl) also significantly improved the storage stability of *L. casei* CRL 431 when compared with bacteria exposed to just one of those stress factors [38].

### 2.5. Prevention of Stress Factors

The prevention of stress factors in the spray drying of probiotic microorganisms can be achieved by careful selection of the appropriate drying strategy [7,13]:

- The addition of protective substances;
- The proper selection of process parameters;
- The adaptation of cells to stress factors before drying.

These preventative measures affect the survival of probiotic bacteria directly during drying, as well as after drying during storage. The following substances are considered protectants and additives used to improve the survival rate of probiotics during spray drying: saccharides, skimmed milk, whey proteins, inulin, trehalose, and oligosaccharides, as well as polymers, such as gum arabic [19,32,33,39,40]. Some examples are also summarized in Table 2. In the scientific literature, several hypotheses have been put forward explaining the protective effect of these substances on the cell membrane and intracellular proteins. These include the theory of vitrification, the hypothesis about the exchange of water molecules in the hydration layer of proteins and the cell membrane with a protective substance (the “water replacement hypothesis”), and the hypothesis of hydration force (the “hydration force hypothesis”) [7].

The activities aimed at optimizing the spray-drying process parameters for probiotic bacteria include, first and foremost, the correct selection of the chamber inlet and outlet air temperatures, the appropriate spray nozzle configuration, the atomizing pressure, and the flow volume of the suspension fed to the dryer [1,19]. A significant improvement in the viability of spray-dried *L. lactis* after replacing air with nitrogen has also been demonstrated [35].

To increase the survival of probiotic cells during spray drying, they can also be exposed to stress conditions during culturing. These stress conditions include exposure to low pH, thermal shock, culturing microorganisms without additional nutrition, exposure to sodium chloride and monosodium glutamate, and culturing with the addition of saccharides, such as mannose and sucrose. In general, it is also believed that cellular biomass derived from the stationary culture phase has better survival rates than that derived from the logarithmic growth phase [20].

There are numerous measures that were developed to improve the viability of freeze-dried bacteria. These include the addition of protective substances to bacterial suspensions, the appropriate control of process parameters during bacteria cultivation, and the adaptation of cells to stress factors before the drying process. The effectiveness of these operations may vary depending on the species of the microorganism [7]. These actions are, in many cases, similar to those used for spray drying.

The most common way to prevent stress factors in freeze-dried probiotic bacteria is to use cryo- and lyoprotectants. Cryoprotectants are water-soluble chemical compounds that lower the melting point of ice. When ice crystals form in the first stage of the process, bacterial cells cluster in the non-frozen fraction. The addition of cryoprotectants increases the volume of the non-frozen fraction of the solution, which increases the space occupied by cells, which in turn reduces cell damage as a result of mechanical and osmotic stresses. These cryoprotectants include polyols, polysaccharides, mono- and disaccharides, amino acids, proteins, minerals, organic acid salts, and complex vitamins [15,41]. In turn, lyoprotectants protect probiotic bacteria at the stage of water removal from the cell. The types and mechanisms of action of lyoprotectants are similar to those described for spray drying. Some sugars, such as sucrose and trehalose, act as both cryo- and lyoprotectants, which translates into their high effectiveness in ensuring the survival of probiotics after freeze drying [22].

The main strategies for protecting probiotic bacteria against stress factors in vacuum drying include the use of protective substances and the selection of process parameters [7]. Among the protective substances most commonly used in the vacuum drying of probiotics are sugars and polyalcohols, such as trehalose and sorbitol. The protective mechanism of these substances is the same as for spray and freeze drying [20].

The relatively low temperature of vacuum drying enables the dehydration of biomass prepared in a semisolid state, such as pellets mixed with a protective substance. The drying efficiency is increased compared to other methods because less water is removed; therefore, a smaller quantity of protective substances can be used [42]. This was proved by vacuum drying (100 mbar, 43 °C, 12 h) *L. bulgaricus* bacteria in the form of a pellet with the addition of powdered lactose, sorbitol, inulin, and xanthan gum [43]. An improvement in the rate of cell survival was found only for samples with 1% sorbitol as the protective substance. The protective effects of sorbitol are due to its ability to lower membrane phase transition temperatures via the interaction with phosphate groups in the membrane [44].

Drying time and temperature are the most important process parameters to be taken into account when optimizing the drying process. The shorter the drying time and the lower the temperature, the higher the survival rate of the dried cells [7]. For example, for *L. delbrueckii* subsp. *bulgaricus* dried at 30, 45, and 70 °C (13.3 mbar, 10 min), damage to the cell membrane was higher with decreases in water activity and increases in drying temperature [42].

The countermeasures used against threats to the viability of fluid-dried bacteria are essentially the same as those used in other drying techniques and include the addition of protective substances, control of process parameters, and the adaptation of cells to stress factors before drying.

The addition of various protective substances to dried probiotic bacteria is the most commonly used protective method. The viability of fluidized-bed-dried probiotic bacteria is also highly dependent on their final humidity. The authors of [1] believed that the critical level of humidity that threatens the viability of *L. helveticus* cells was 1–3%. It was indicated that this might depend on the bacterial species; for *L. salivarius*, for example, the critical humidity was in the range of 5–6%.

There are also certain factors that can influence the viability and survival of probiotic bacteria during storage. To increase the shelf-life of dried probiotics, various protective measures can be used. These include the addition of antioxidants, such as 0.5% (*w*/*w*) vitamin E; they protect the final formulation against oxidative stress [38]. Storage at a lower temperature, i.e., 4 °C, can also result in an improvement in the survival rate of dried probiotic powder compared to the results when stored at 22 °C or 35 °C [22]. Examples of shelf-life analyses and viability measurements during storage are summarized in Table 3.

**Table 1:** Comparison of cell concentrations after different drying methods.

Reduction [log cfu/g]	Microorganism	Growth Parameters	Cell Concentration before Drying [log cfu/g]	Cell Concentration after Drying [log cfu/g]	Drying Method	Reference
<1	*Lactiplantibacillus plantarum 299v*	MRS broth, 37 °C	10.3	11.3	Freeze drying	[16]
	*Pediococcus acidilactici HA-6111-2*	MRS broth, 37 °C	10.5	11.2	Freeze drying	[16]
<1	*Lactiplantibacillus plantarum 299v*	MRS broth, 37 °C	9.4	9.5	Spray drying	[16]
	*Pediococcus acidilactici HA-6111-2*	MRS broth, 37 °C	9.0	9.4	Spray drying	[16]
	*Lactobacillus kefir* CIDCA 8348	MRS broth, 30 °C	8.8	8.2	Spray drying	[45]
	*Lactobacillus plantarum* CIDCA 83114	MRS broth, 30 °C	9.9	9.8	Spray drying	[45]
	*Lactobacillus kefir* CIDCA 8321	MRS broth, 30 °C	8.4	8.1	Spray drying	[45]
	*Lactobacillus rhamnosus LGG*	MRS broth, 37 °C	11.0	10.2	Spray drying	[46]
	*Lactobacillus casei AMBR2*	MRS broth, 37 °C	11.0	10.3	Spray drying	[46]
>1	*Lactobacillus acidophilus* NCDC016	MRS broth, 37 °C	11.2	10.0	Spray drying	[32]
	*Escherichia Coli* K12	TSB, 30 °C	10.7–10.9	7.9	Spray drying	[31]
>1	*Lactobacillus reuteri* DSM 20016	MRS broth, 37 °C	8.7–9.7	7.7	Fluidized bed drying	[47]

**Table 2:** Protective effects of different materials.

Reduction Post-Drying [log cfu/g]	Microorganism	Drying Method	Protective Substances	Cell Concentration before Drying [log cfu/g]	Cell Concentration after Drying [log cfu/g]	Survivability [%]	Reference
<1	*Bifidobacterium bifidum*	Spray drying (double layered)	Gum arabic 9%, 1% β-cyclodextrin, 1% lecithin	6.93	6.18	89.22	[48]
	*Bifidobacterium bifidum*	Spray chilling (double layered)	Hydrogenated palm oil, 2% Tween 80	6.12	6.01	98.25	[48]
	*Saccharomyces cerevisiae* var. *boulardii*	Spray drying	Gelatin 10%	9.95	9.06	91.55	[49]
	*Saccharomyces cerevisiae* var. *boulardii*	Spray drying	Whey protein concentrate 20%	9.65	8.86	91.81	[49]
	*Lactobacillus rhamnosus*	Spray drying	Native rice starch 10%	9.26	8.98	53.24	[40]
	*Lactobacillus rhamnosus*	Spray drying	Inulin 15%	9.18	8.91	53.55	[40]
<1	*Lactobacillus brevis* WK12	Freeze drying	Soy powder solution 10%	11.30	11.26	90.00	[50]
	*Lactococcus lactis* WK11	Freeze drying	Soy powder solution 10%	11.30	11.27	94.00	[50]
>1	*Bifidobacterium bifidum*	Spray drying	Gum arabic 9%, 1% β-cyclodextrin	10.12	7.57	74.81	[48]
	*Bifidobacterium bifidum*	Spray chilling	Hydrogenated palm oil, 2% Tween 80	9.51	8.25	86.79	[48]
	*Saccharomyces cerevisiae* var. *boulardii*	Spray drying	Modified starch 20%	9.65	8.64	89.53	[49]
	*Saccharomyces cerevisiae* var. *boulardii*	Spray drying	Maltodextrin 20%	9.65	8.61	89.24	[49]
	*Saccharomyces cerevisiae* var. *boulardii*	Spray drying	Pea protein isolate 10%	9.95	8.55	86.52	[49]
	*Saccharomyces cerevisiae* var. *boulardii*	Spray drying	Gum Arabic 20%	9.65	8.17	84.69	[49]

**Table 3:** Shelf-life and viability of probiotics.

Microorganism	Preparation Method and Matrix	Storage Conditions	Initial Cell Concentration [log cfu/g]	Cell Concentration after Storage [log cfu/g]	Monitored Parameters	Reference
*Bacillus coagulans*	Fluid-bed-dried apple snacks	90 days at 25 °C	7.89	6.78	viable cell counts, water activity and moisture content, enzyme activity, total phenolic content, antioxidant capacity, vitamin E concentration	[29]
*Lactiplantibacillus plantarum* 299v	Spray drying in orange juice	12 months, 25 °C, a_w_ = 0.03 hermetic glass flasks with silica gel	7.90	6.30	viable cell counts, water activity	[51]
*Pediococcus acidilactici* HA-6111-2	Spray drying in orange juice	12 months, 25 °C, a_w_ = 0.03 hermetic glass flasks with silica gel	8.70	8.00	viable cell counts, water activity	[51]
*Lactiplantibacillus plantarum* Lp 115-400b	coconut water oatmeal with inulin (1 g/100 mL)	4 °C, 49 days	7.06 (9.12 at day 7)	7.23	viable cell counts, pH, lactic acid content, rheological parameters	[52]
*Lactiplantibacillus plantarum* Lp 115-400b	coconut water oatmeal	4 °C, 49 days	6.99 (9.01 at day 7)	6.41	viable cell counts, pH, lactic acid content, rheological parameters	[52]

## 3. New Trends in the Drying and Application of Probiotics

Until recently, the standard methods of preserving probiotic bacteria consisted of the four types of drying presented above (spray drying, freeze drying, vacuum drying, and fluid bed drying). As new technologies and needs emerge, we observe the development of new trends in the drying and formulation of probiotics. These include new application methods that combine the existing methods, research on new matrices for probiotic bacteria, and new properties of probiotic strains.

### 3.1. Various Application Methods

New developments in the formulation of dried probiotics include different forms of administration, such as nasal sprays and creams or lotions. The development of new application methods for probiotics, besides orally administered tablets and microcapsules, allows for better use of their wide range of health-promoting properties. It also allows them to be better adapted to the needs of patients.

Probiotic formulations have been found to have a beneficial effect on the upper respiratory tract (URT), preventing acute respiratory tract infections. *L. casei* AMBR2, isolated from the human URT, was chosen for the preparation of a probiotic nasal spray by spray drying [4]. For the preparation of the drying solution, different combinations of saccharides and polymers were used. The following combinations provided the highest shelf-life stability for *L. casei* AMBR2 while also maintaining a stable formulation: 2.5% (*w*/*v*) sucrose and 0.4% (*w*/*v*) xanthan gum, 2.5% (*w*/*v*) isomalt and 0.4% (*w*/*v*) xanthan gum, 2.5% (*w*/*v*) trehalose and 1% (*w*/*v*) HPMC (hydroxypropyl methylcellulose), and 2.5% (*w*/*v*) lactose and 1% (*w*/*v*) HPMC. Shelf-life viability was measured during 28 weeks of storage under refrigerated conditions, and all mentioned formulations showed a viability level higher than 5 × 10^9^ CFU/g. During the spray tests, after the resuspension of the powder, no significant viability changes were noticed. In the course of the functionality tests, adherence to the Calu-3 cell line was measured. For the formulations containing HPMC, adherence was unchanged in comparison to the adherence of fresh *L. casei* AMBR2 cells (>10%). Formulations containing xanthan gum had significantly lower levels of adherence (up to 5%). The antimicrobial effects of *L. casei* AMBR2 against *S. aureus*, *M. catarrhalis*, and *H. influenzae* (URT pathogens) were also confirmed for both fresh and dried cells, with lower inhibition zones in some assays with dried samples.

Topically applied probiotics are the focus of many studies as the combination of probiotic and antimicrobial properties allows for the effective treatment of different skin disorders, including atopic dermatitis and acne. Atopic dermatitis is a chronic, inflammatory skin condition traditionally treated with histamines, corticosteroids, biopharmaceuticals, and antimicrobials. Other non-pharmaceutical methods of treatment are being researched because, for some patients, the currently used treatments are ineffective or associated with side effects. A lotion containing the heat-treated probiotic strain *Lactobacillus johnsonii* NCC 533 was applied for 3 weeks in a group of patients with atopic dermatitis [5]. The results obtained after the treatment showed that the probiotic lotion controlled *Staphylococcus aureus* colonization (which is one of the causes of atopic dermatitis, especially in the acute phase). Additionally, a local clinical improvement was found in patients who used the lotion over 3 weeks according to SCORAD (the SCORing Atopic Dermatitis tool).

Antimicrobial activity is also important in the treatment of other skin disorders, such as acne. The skin adhesion and antimicrobial activity of different probiotic strains were studied by Lopes et al. [53]. The use of probiotics allows for the equilibration of the skin microbiota and modulates the immune system. Additionally, the bacteriocins produced by certain probiotic strains affect pathogenic microorganisms by inhibiting their growth. Cell-free culture supernatants (CFCS) were used for evaluation of this phenomenon and showed antimicrobial activity towards *Escherichia coli*, *Cutibacterium acnes*, *Staphylococcus aureus*, and *Pseudomonas aeruginosa*. Their antimicrobial activity was confirmed by measuring the inhibition zones. The observed effect was attributed to the production of organic acids by lactic acid bacteria, which were able to inhibit the growth of pathogenic microorganisms by decreasing the pH of the medium. In tests with neutralized cell-free culture supernatant, no antimicrobial activity was observed; this result further proved that, in this case, the antimicrobial effect was linked with the production of organic acids and not with the production of bacteriocins. Most of the used strains also displayed the ability to prevent biofilm formation by reducing the ability of pathogenic cells to attach to and create a biofilm. Among seven tested probiotic strains, three were able to prevent biofilm formation by *E. coli*, five of them prevented biofilm formation by *S. aureus*, six prevented biofilm formation by *P. aeruginosa*, and all strains showed the ability to decrease biofilm formation by *C. acnes*.

### 3.2. Controlled Release

Controlled release is one of the main focus points in the current development of drugs, biopharmaceuticals, and supplements. In addition, for probiotics, it is key to prepare formulations that are capable of withstanding the acidic environment and various enzymes found in the gastrointestinal tract while retaining their properties and viability for release in the intestine.

Sánchez-Portilla et al. [21] used polymethacrylate-based copolymers (Eudragit) to prepare microencapsulated *Bifidobacterium* by fluid bed drying. Two matrices were used in this process: microcrystalline cellulose (Avicel) and prebiotic inulin. During preparation, the viability of the cells decreased from the initial concentration of 10^9^ by 0.99 log_10_ cfu/g for the formulation with Avicel and by 1.33 log_10_ cfu/g for the formulation with inulin. During long-term storage, viability was maintained at 6.6 log_10_ cfu/g for the first 3 months and remained at over 4.5 log_10_ cfu/g for both formulations after 2 years of storage. During the resistance tests, both products resisted stomach acidic conditions of pH 3. The main difference between the two products was in the mechanism of the release of *Bifidobacteria*. In the product with inulin as a matrix, due to the water solubility of inulin, release was dependent only on the protective polymer used for coating. Meanwhile, in the product with microcrystalline cellulose as a matrix, release was dependent on both the coating layer and the matrix particles, which retained the bacteria and provided resistance even under alkaline conditions.

Targeted release was also the goal of the study conducted by Huang et al. [54]. Alginate, sucrose, whey protein isolate, and shellac were used as encapsulating materials in the external emulsification process, after which the microcapsules were freeze-dried. *L. reuteri* TMW 1.656 was exposed to different stress conditions (digestive and gastric juices as well as heating) after microencapsulation and drying; then, it was stored under ambient conditions to measure the inactivation of the cells. Probiotic stability was higher for the alginate capsules with added shellac under all conditions, which was explained by the decrease in hygroscopicity. The addition of whey protein isolate to alginate and shellac resulted in a further increase in viability (especially for gastric juice exposure and heat shock), and this finding was related to the protective effects of whey protein. The combination of modified rice protein and shellac was used by Wang et al. (2021) [55] to prepare an enteric coating for probiotic microcapsules. The addition of modified rice protein was able to modify the properties of shellac-based coatings. The results showed that the version with the addition of rice protein was superior at preserving probiotic viability during storage and digestion. Similarly, microcapsules composed of sporopollenin exine and coated with calcium alginate/carboxymethylpachymaran shell were confirmed to enhance the storage stability of probiotics while providing sustained release in the gastrointestinal tract [56].

Interpenetrating polymer network (IPN) hydrogels obtained by the enzymatic method were studied by Yan et al. [57] as potential carriers for probiotics. The hydrogels were prepared using a combination of biopolymers, namely soy protein isolate and sugar beet pectin. The concentrations of soy protein isolate in the final solution varied from 4% to 10%, and the sugar beet pectin concentration varied from 2.5% to 5%. Afterwards, a probiotic suspension containing about 10 log cfu/mL *L. paracasei LS14* was added and mixed with the hydrogel solution. To improve hydrogel formation, the enzyme laccase was also added in different concentrations (2–14 U/g). Hydrogel containing 10% SPI and 3.5% SBP and induced by 10 U/g laccase was highlighted as yielding the highest probiotic viability (~7.5 cfu/mL). It was also the least sensitive to simulated gastric conditions, showing no decrease in probiotic cell viability after 2 h of incubation. 

### 3.3. Probiotics in Food Matrices

Probiotics are available in a wide array of commercial products, but most of them are offered in the form of dietary supplements. However, there is a large group of products that contain natural probiotics, such as naturally fermented yogurts, kefir, kimchi, and sauerkraut [58]. In addition, non-fermented and, more importantly, non-dairy food products can provide a matrix for probiotics. The use of different food matrices allows for the preparation of healthy probiotic food, taking into account allergies, nutritional preferences, taste, and the aroma of the final product. Probiotic food products of non-dairy origin, such as cereals, fruit, and vegetable- and meat-based products, are also rich sources of protein, minerals, vitamins, dietary fiber, antioxidants, and other bioactive substances that may provide extra health benefits [59]. Another advantage of using food matrices is their potential to improve the storage stability of probiotics.

Chocolate is one of the most versatile functional foods, and it can be enriched with probiotics without the loss of sensory attributes [60]. It can also be prepared in various forms, and it can combine different beneficial effects. For example, chocolate and hazelnut spreads were developed that are not only enriched with probiotics but also contain less fat, which was replaced by healthy triacylglycerols [61]. There are, however, some challenges to overcome, such as processing, storage, and gastrointestinal conditions. Formulations containing cocoa powder and sodium alginate were used in the encapsulation of probiotics by emulsion-based freeze drying, with an encapsulation efficiency of up to 95% [62]. The use of encapsulated probiotics allowed researchers to maintain the viability of the probiotics in the product at over 7.5 log cfu/g after 90 days of storage at 25 °C; they also recorded a high level of viability (8.0 log cfu/g) during in vitro gastrointestinal digestion.

Among the various products with probiotic properties is butiá (*Butia odorata)* ice cream supplemented with *Bifidobacterium lactis* (BI-04). Ice cream ensured the viability of probiotic bacteria during 90 days of storage at −18 °C. Additionally, butiá was able to maintain its bioactive components during storage. The authors also conducted a market analysis that showed high acceptance of novel functional food products among panelists [6].

Banana powder is an excellent probiotic matrix due to its porous structure, nutritious properties, low cost, and overall availability. Freeze-dried banana powder was prepared after mixing banana paste with different probiotic formulations. These formulations included *Lactobacillus acidophilus* and *Lactobacillus casei* microencapsulated with whey protein isolate, fructooligosaccharides, and a combination of both at a 1:1 ratio. After the incorporation into banana paste, the probiotic formulations were then refrigerated for 72 h at −32 °C and transferred to a freeze dryer for 24 h at −40 °C. Afterwards, the obtained product was ground and filtered into a fine powder. The results show that the use of microspheres combining whey protein isolate and fructooligosaccharides resulted in the highest encapsulation yield (98%). The obtained product was also stable during storage at the temperature of 4 °C and increased the viability of bacteria after 90 min of exposure to gastrointestinal conditions (7.85 log CFU/g and 7.52 log CFU/g for the microencapsulated cells of *L. acidophilus* and *L. casei*, respectively) compared with free cells (a decrease of 4.69 log CFU/g for *L. acidophilus* and 5.64 log CFU/g for *L. casei*) [63].

The survival of microencapsulated *Lactococcus lactis* Subsp. *lactis* R7 was measured for various food matrices by Rosolen et al. [64]. A quantity of 1 g of spray-dried bacteria microcapsules containing approximately 12 log CFU g^−1^ was added to 100 mL of milk, milk cream, and blueberry juice. These samples were then stored for 28 days at 4 °C and compared with samples containing free, non-encapsulated bacteria. The results showed that the acidic pH of blueberry juice had damaging effects on free cells, resulting in their viability falling below the minimum value to be considered probiotic after 14 days of storage. The microencapsulated sample had better viability in the blueberry juice, showing a loss of viability after 21 days. In both milk and milk cream, the microencapsulated bacteria showed higher levels of viability after 28 days of storage than the samples with free cells. Additionally, the free cells promoted higher acidification in milk, which proves that the probiotic bacteria were successfully trapped inside the microcapsules during encapsulation.

## 4. Assessment of Strain Suitability and Viability

While almost all probiotic formulations use lactic acid bacteria with GRAS status, there are some additional requirements to be met when preparing a probiotic product. Different strains can vary in terms of their environmental resistance, growth characteristics, production of metabolites, and their effects on human health, which can make some more suitable for certain applications. This assessment should be conducted using both classic microbiological methods and more advanced tools, such as flow cytometry, while also including a thorough analysis of gene expression and proteomics. Table 4 lists methods used in recent studies to assess probiotic bacteria.

### 4.1. Microbiological Analysis

The basic procedure in the assessment of probiotics is the use of plate cell counts for the enumeration of viable cells. It can be divided into the following steps [65]:

1. Sample preparation: This depends on the matrix (frozen, dried, liquid, or free cells).
2. Dilution: This includes the prior homogenization or rehydration and the use of a dilution medium containing peptone, NaCl, or phosphate salts, as well as the addition of antioxidants for oxygen-sensitive strains.
3. Plating: This is performed with a strain-specific plating medium.
4. Incubation: This takes into consideration the optimum temperature (mostly 37 °C as many probiotics naturally inhabit the gastrointestinal tract) and the aerobic/anaerobic conditions preferred by the specific strain.

One of the issues with relying solely on plate counts for viability assessments of probiotics is that such counts are only limited to the viable cells growing on the plate medium. There are, however, other groups of cells, such as those that are viable but not culturable (VBNC), that should be considered in the assessment of probiotics [66].

### 4.2. Flow Cytometry

Flow cytometry can be used to expand the assessment conducted using conventional microbiological methods. The viability of probiotic cells can be analyzed using dual staining with SYBR Green I and propidium iodide (PI) dyes. SYBR Green I allows the detection of viable and active cells by binding to DNA and emitting green light, while PI indicates cellular damage in dead cells because it is membrane impermeable and only binds to DNA in cells with damaged membranes, emitting red light. Such a protocol enabled the identification of viable and dead cells in *L. plantarum* [67] and allowed for a much faster analysis compared to conventional microbiological methods while providing a higher number of observations and, thus, increasing statistical certainty.

### 4.3. Gene Expression and Proteomic Analysis

Despite the wide application of probiotic bacteria and their extensive use, some mechanisms, especially those linked with stress responses, remain to be addressed. Researching the proteins and gene expression involved in these processes can allow for a better understanding of the stress responses to drying and environmental conditions, allowing for better cell survival and industrial process optimization.

The protein expression patterns of *Oenococcus oeni* SD-2a that was subjected to freeze-drying stress were analyzed by Yang et al. [68]. Biofilm formation is the main method of adaptation of *O. oeni* against stress and is linked with different potential signals (QS autoinducers, peptides, and volatile and organic compounds), matrix composition (polysaccharides), and energy supply (carbon starvation). The HSP20 protein and Clp proteases were also linked with improved resistance to stress induced by the addition of monosodium glutamate (MSG), which improved cell integrity during freeze drying.

*Leuconostoc mesenteroides* BD3749 forms cell aggregates as a reaction to oxidative stress through the upregulation of the glucansucrase-encoding gene Gsy. *L. mesenteroides* BD3749 cells synthesize large amounts of insoluble exopolysaccharides in response to oxidative stress, which reduces the accumulation of reactive oxygen species in bacterial cells, improving their survival. In a relevant study, Gsy and its upregulation following exposure to oxygen were responsible for the synthesis of those insoluble exopolysaccharides that mediated the aggregation processes [69].

**Table 4:** Methods for the assessment of probiotics.

Method	Key Applications	Reference
Plate counting	viable cell enumeration	[65]
Flow cytometry	cell integrity, membrane damage (live/dead staining)	[46,67]
Scanning electron microscopy	cell morphology, surface characteristics	[46,68]
Laser diffraction	particle size	[46]
Two-dimensional gel electrophoresis (2-DE)	protein pattern analysis (proteins involved in biofilm formation, quorum sensing, volatile compounds production, stress response)	[68]
Mass spectrometry	peptide mass fingerprinting	[68]
Bioinformatics	protein identification, prediction of protein interaction, subcellular localization	[68]
RT-qPCR	gene expression	[69]

## 5. Future Challenges and Concluding Remarks

**Drying techniques:** Among the methods described, freeze drying guarantees the best viability of dried bacteria. It is, however, the most expensive method, and the process has a long duration. In those two categories, both fluid bed drying and spray drying are better, with fluid bed drying being less expensive and faster than spray drying. Fluid bed drying also has additional potential as a secondary method, i.e., it can be combined with other drying techniques to coat the product in a fluid bed after drying using a different method, further improving its stability during storage. Newly developed methods and modifications of known techniques, such as electrospinning and spray freeze drying, offer opportunities for further technological development and could potentially replace the techniques used up until now.

**Response to stress conditions:** It has been pointed out that the influence of different stresses associated with drying on the viability of the cells can be decreased by habituating those cells to stress factors in the culturing stage. Such methods have not yet been thoroughly studied in relation to gene expression. Knowledge of the expression of specific genes in response to targeted stress may be beneficial for the production of stable and viable probiotics with longer shelf-life.

**Multiparametric analysis:** Most studies focus on the survival of bacterial cells during drying. While this is an important parameter, it is not ideal for the assessment of the quality of the obtained product. It does not take into consideration the activity of the probiotics in the gastrointestinal tract, which may be influenced by the type of drying, the drying conditions, the use of protectants, and the stresses experienced by cells in the digestive tract. Other parameters worth researching are cell activity (and the activity of viable but nonculturable cells), cell adhesion to the intestinal epithelium, and the expression of shock proteins.

**Strain viability:** It should also be noted that the vast majority of research currently focuses on a poorly diversified group of bacteria, mostly lactobacilli. Finding new diverse strains with probiotic activity should be a focus of future studies. The differentiation of probiotics could lead to the development of new formulations targeting various health issues at once. It could also help to develop probiotics as food additives with strains conferring positive effects both in terms of their probiotic activity and possibly also antimicrobial activity. There is also an increasing tendency in the market to develop multi-strain probiotics. Such products offer a broader spectrum of beneficial and synergistic effects. However, such effects are not yet well studied and need future work [70].

**New applications:** Probiotics have taken many new and varied forms. The development of products suitable for use in different applications should not be focused only on dietary supplements and dairy probiotic food, which have been the point of focus of many studies conducted on probiotics. New applications are being sought in fields such as cosmetics, functional foods, non-dairy probiotic foods, nutraceuticals, and medicine [46,59,71].

**Target groups:** Following global trends should be a priority when selecting target groups for probiotics. For example, allergies are an increasing public health concern; therefore, measures to mitigate or eliminate their effects are being researched. The beneficial effects of probiotics have been proven in rhinitis, asthma, and atopic dermatitis [72]. Another global trend, especially pronounced in developed countries, is the aging of society. Probiotics enhance the immune response, which is especially important in elderly individuals. Additionally, probiotics may improve the effectiveness of vaccination [73].

## Data Availability

Data is contained within the article.
